# Acute Ischemic Stroke in Pregnancy: A Practical Focus on Neuroimaging and Reperfusion Therapy

**DOI:** 10.1007/s00062-022-01215-5

**Published:** 2022-09-16

**Authors:** Marcin Wiącek, Antonina Oboz-Adaś, Katarzyna Kuźniar, Anna Karaś, Patryk Jasielski, Halina Bartosik-Psujek

**Affiliations:** 1 Institute of Medical Sciences, University of Rzeszow, Rzeszow, Poland; 2 Department of Neurology, Clinical Regional Hospital No. 2, Rzeszow, Poland

**Keywords:** Stroke, Gestation, Mechanical thrombectomy, Intravenous thrombolysis, Computed tomography

## Abstract

**Introduction:**

Pregnancy increases the risk of acute ischemic stroke (AIS) among young women and is responsible for about 5% of maternal deaths and significant disability. Concerns of potential adverse events of imaging and reperfusion therapies in this group of patients can lead to a substantial delay or omission of treatment that can significantly worsen outcomes.

**Objective:**

The objective of this study is to discuss main concerns of diagnosis and therapy of pregnant patients with AIS regarding neuroimaging and reperfusion treatment.

**Results:**

The cumulative radiation dose of computed tomography (CT)-based entire diagnostic procedure (noncontrast CT, CT-angiography and CT-perfusion) is estimated to be below threshold for serious fetal radiation exposure adverse events. Similarly, magnetic resonance imaging(MRI)-based imaging is thought to be safe as long as gadolinium contrast media are avoided. The added risk of intravenous thrombolysis (IVT) and mechanical thrombectomy during pregnancy is thought to be very low. Nevertheless, some additional safety measures should be utilized to reduce the risk of radiation, contrast media and hypotension exposure during diagnostic procedures or reperfusion treatment.

**Conclusion:**

Fetal safety concerns should not preclude routine diagnostic work-up (except for gadolinium contrast media administration) in childbearing AIS women, including procedures applied in unknown onset and late onset individuals. Due to rather low added risk of serious treatment complications, pregnancy should not be a sole contraindication for neither IVT, nor endovascular treatment.

## Introduction

Stroke is the second cause of long-term disability and death worldwide [1]. Its prevalence is projected to rise from 1.1 million in 2000 to 1.5 million per year by 2025 in Europe [2]. Despite being a rather rare event in women of childbearing age (10 per 100,000 women) its incidence can increase to over 30 cases per 100,000 during pregnancy and peripartum. Being responsible for about 5% of maternal deaths and a substantial disability rate, the burden of stroke during pregnancy is unquestionably high [3, 4].

It is now known that acute ischemic stroke (AIS), with timely employment of reperfusion therapy, can be a highly treatable condition [5, 6]; however, pregnant women were excluded from all of the randomized controlled trials (RCTs) confirming efficacy of both intravenous thrombolysis (IVT) and mechanical thrombectomy (MT) for the reason of maternal and fetal safety. Therefore, no optimal treatment in this group of patients has been defined [7]. In addition, the tremendous progress in neuroimaging procedures, such as introducing of perfusion techniques (computed tomography perfusion, CTP, magnetic resonance perfusion, MRP) and their employment in the treatment qualification process, has created additional diagnostic difficulty. Some authors highlight that the concern of potential adverse events of imaging and reperfusion therapies can lead to a substantial delay or omission of treatment [8]. It all makes the timely AIS diagnostic and management decisions during pregnancy very challenging. The rapid treatment risk and benefit assessment is therefore of great importance.

This review aims to summarize the current state of knowledge and proposes a practical approach to acute brain imaging and treatment of AIS during pregnancy.

## Early Neuroimaging in Pregnancy

Neuroimaging procedures are essential in AIS reperfusion therapy qualification process. In the general population both computed tomography (CT) and magnetic resonance imaging (MRI) are effective in excluding intracerebral hemorrhage (ICH) and the preferred method is chosen based on its local availability to ensure rapid treatment introduction. Similarly, endovascular treatment (EVT) eligibility assessment based on noninvasive cerebral vessel imaging can interchangeably utilize MR angiography (MRA) or CT angiography (CTA) [7]. In pregnancy, not only the pace of the qualification process, but also fetal safety should be taken into consideration when selecting imaging techniques.

There is also a group of AIS patients beyond the standard time window for reperfusion therapy that could potentially benefit from employment of advanced neuroimaging techniques, such as CTP or MRP imaging. This approach has not been validated in stroke during pregnancy and will be a subject of our theoretical considerations.

### Initial Noncontrast Imaging

Noncontrast head CT poses a theoretical risk of fetal adverse events due to radiation exposure; however, its estimated X‑ray dose to an unborn child ranging between 0.001 and 1 mSv is thought to be very low [9, 10]. As a comparison, the expected cumulative dose of background radiation absorption during the entire pregnancy is about 1 mSv and the typical occupational limit for fetal radiation equals 5 mSv [11, 12]. Animal studies showed the risk of prenatal death due to irradiation to be highest during the preimplantation period (0–2 weeks) and of congenital anomalies or growth restriction during organogenesis (2–8 weeks). The approximated thresholds for those events are 50–250 mSv. Radiation exposure associated with the significant risk of severe intellectual disability (60–310 mSv) is thought to be much higher than the amount absorbed by fetus during head CT as well [11, 13]. In fact, there was no abortion, growth restriction or anomalies reported with levels below 50 mSv [9]. The risk of carcinogenesis is not well evaluated but considered to be very low in case of radiologic examinations above the diaphragm and below the knees [9]. It rises with the amount of exposure and is estimated to elevate the background rate by less than 1 in 10,000 cases with doses < 1 mGy [14, 15] and 1 in 2000 with 10–20 mSv [16, 17]. Given the information above, it should be concluded that the fetal ionizing radiation exposure from noncontrast head CT is probably negligible and concerns of its safety must not preclude prompt utilization of this imaging modality in cases of maternal stroke; however, additional safety measures, such as shielding of the abdomen/pelvis and minimizing scans should be routinely incorporated [12].

The use of MRI eliminates the roentgen irradiation with all of its potential adverse events. Studies have also failed to show increased risk of stillbirth, neonatal death, neoplasm or congenital abnormalities in infants whose mothers were exposed to MRI during the first trimester [18]. There are no known biological effects on fetuses during later periods of pregnancy as well [19]. Therefore, in childbearing women it is considered to be the neuroimaging method of choice [19, 20]. Its downsides include longer than CT scan duration and limited availability in some centers.

In conclusion, in case of disabling stroke during pregnancy rapid neuroimaging utilizing the readily available method (CT or MRI) should be performed. When both imaging modalities may be applied without significant delay or reperfusion treatment is not considered, MRI should be preferred over CT.

### Noninvasive Cerebral Vessel Imaging

Noninvasive vessel imaging should be a part of routine initial AIS evaluation. Its primary objective is to assess for large vessel occlusion (LVO) as a part of EVT qualification process. For this purpose CTA and MRA are considered to be equivalent methods, with the former having slightly higher accuracy than the latter [7].

During CTA iodinated contrast agent is applied that may bring some fetal safety concerns. Despite crossing the placenta via simple diffusion, in vitro studies have failed to show mutagenic effect of iodinated contrast media and teratogenic effect has not been observed in animal studies [21–24]. Nevertheless, there were no such studies performed in pregnant women and most of iodinated contrast agents are classified as class B by the U.S. Food and Drug Association (FDA) [25]. There was one study providing data on neurodevelopment after iodinated contrast, which was normal until the last follow-up at the age of 4 months [26].

Administration of iodinated contrast media in pregnancy may result in goiter formation and underactive thyroid in neonates, although studies that demonstrated those side effects were performed with liposoluble compounds during amniofetography or hysterosalpingography [27]. CTA utilizes water-soluble non-ionic media that are considered to have less adverse effects [28]. In the systematic review of studies including 525 neonates born after maternal contrast agent exposure during CT scanning in pregnancy, the overall proportion of (transient) neonatal thyroid dysfunction was estimated at 0% (95% CI: 0.0–0.02% I^2^ = 0%). Transient thyroid dysfunction was found in only 3 out of 525 (0.6%) neonates and resolved spontaneously in all of them [29]. It is also worth noting that childbearing women with a history of allergic reactions have an increased risk of developing an allergic reaction during pregnancy that may pose some risk for the fetus [27].

The American College of Obstetricians and Gynecologists stated that despite the lack of known harm, it is generally recommended that contrast only be used if absolutely required to obtain additional diagnostic information that will affect the care of the fetus or woman during the pregnancy [19]. In cases of disabling AIS the requirement of obtaining additional important information, i.e. presence of the LVO, is certainly met.

Magnetic resonance angiography techniques used in AIS patients include non-contrast time-of-flight MRA (TOF-MRA) and contrast-enhanced MRA (CE-MRA). Despite several disadvantages (e.g. limited field of view that excludes extracranial vessels, long acquisition time with the risk of motion artifacts) TOF-MRA is more widely used in EVT qualification process [30]. CE-MRA can be superior to TOF-MRA in terms of localizing vessel occlusion and accuracy of collateral status assessment. It also offers shorter acquisition time and larger coverage [31, 32]; however, it requires gadolinium contrast administration that addresses questions about its safety in pregnancy.

Animal investigations on teratogenic effect of gadolinium-based contrast media have been inconclusive, with some studies showing high and repeated doses to be teratogenic, while others reported no such effects [27, 33]. Understandably, no trials on pregnant women were conducted. Retrospective studies have presented no significant risk of congenital abnormalities amongst neonates whose mothers were exposed to gadolinium-based contrast [18, 34]. One large retrospective study assessing long-term safety of MRI exposure during the first trimester of pregnancy showed the risk of rheumatological, inflammatory or infiltrative skin conditions to be increased in gadolinium MRI group compared to no MRI (adjusted hazard ratio, 1.36; 95% CI 1.09–1.69). There was also significantly higher risk of stillbirth and neonatal death in 7 gadolinium MRI-exposed versus 9844 MRI unexposed pregnancies (adjusted relative risk, RR 3.70; 95% CI 1.55–8.85) [18]. Despite several shortcomings of this study, it may bring the conclusion that MRI with gadolinium contrast enhancement should be used with the highest caution. According to the guidelines of American College of Obstetricians and Gynecologists it should be limited to the instances when it significantly improves diagnostic performance and is expected to improve fetal or maternal outcome [19]. Given the possibility of TOF-MRA utilization, CE-MRA should not be used as a method of noninvasive cerebral vessel assessment in EVT patient selection during pregnancy.

Considering the abovementioned data and uncertain benefits of EVT treatment of minor AIS, it is reasonable to employ a preimaging selection method based on admission National Institutes of Health Stroke Scale (NIHSS) in pregnant stroke patients. The NIHSS score was found to be the best instrument for LVO prediction, with a threshold of ≥ 6 having the highest sensitivity (87%) and modest specificity (52%). This approach is a valid option in the general population according to AHA/ASA treatment guidelines and might be also reasonable among childbearing women [7]. A more conservative cut-off NIHSS value of ≥ 10 would provide higher specificity (74%) at the cost of lower sensitivity (73%) [7, 35].

According to the Canadian Stroke Best Practice Consensus Statement on acute stroke management during pregnancy, in selected cases (e.g. presence of a hyperdense middle cerebral artery on CT), direct digital subtraction angiography (DSA) for potential treatment of LVO may be performed instead of initial noninvasive assessment [12]. Considering the high specificity of the hyperdense artery sign, this may be a rational approach [36].

In conclusion, TOF-MRI appears to be the safest noninvasive cerebral vessel imaging method. It should be incorporated into the pregnant AIS patient assessment for LVO when MRI was chosen as the initial imaging modality. It is also reasonable to avoid using CE-MRA in this group of patients. In case of CT being initially selected, CTA should be performed as its fetal adverse effects appear to be negligible. In our opinion, the choice between CT with CTA and MRI with MRA should preferably be made based on availability and pace, rather than safety concerns. It appears reasonable to employ ≥ 6 (or more conservative ≥ 10) NIHSS score threshold for noninvasive cerebral vessel imaging and to abandon the examination in cases of pregnant women otherwise ineligible for EVT.

### Perfusion Imaging and Late Presenting or Unknown Onset AIS Patients

A subset of late presenting stroke patients benefits from reperfusion treatment when appropriately selected [7]. Several selection methods have been evaluated and are now widely employed. AIS patients with LVO presenting between 6 and 24 h from symptom onset are good candidates for EVT as long as they have favorable clinical radiological mismatch profile, where diffusion-weighted imaging (DWI) MRI sequences serve as a radiological and NIHSS score as a clinical component according to DAWN trial criteria [37]. In the 6–18 h window CTP and MRP may serve the same purpose as shown in DEFUSE‑3 trial [38]. Recently, CLEAR study showed no significant differences in outcomes with non-contrast CT only compared with CTP or MRI-based patient selection, which may bring new insights into the previous EVT qualification paradigm [39]. Similarly, IVT may be offered to a subset of late presenting patients or those with unknown stroke onset. According to European Stroke Organization (ESO) guidelines, patients with AIS of 4.5–9 h duration and CTP/MRP mismatch or those with symptoms on awakening and MRI DWI fluid inversion recovery (FLAIR) mismatch are good candidates for IVT [40]. It shows several clinical scenarios in which childbearing women with AIS may be found. None of them have been assessed in clinical trials, as pregnancy was an exclusion criterion in all of them. Considering that perfusion studies play a pivotal role in most of those scenarios, their safety for an unborn child should be taken into consideration.

Depending on the study, additional effective radiation dose for CTP varies between 0.2 and 9 mSv [41–43]. The cumulative dose of non-contrast CT, CTA and CTP was estimated to be 11.48 mSv, but can be reduced by nearly 50% using some low-dose protocols [43, 44]. There are no studies evaluating CTP radiation dose absorbed by fetus, but it is probably low, as local organ dose for gonads was shown to equal 0.2 mSv [42]. The threshold for radiation exposure adverse events is thought to be much higher than the amount absorbed during entire AIS CT evaluation (non-contrast CT, CTA, CTP). The risk of additional contrast injection has not been evaluated but is probably negligible as described in the “Noninvasive cerebral vessel imaging” section. In late presenting or unknown onset pregnant patients with disabling stroke, CTP may bring some vital information that would potentially lead to reperfusion treatment. Therefore, its utilization in selected patients appears to be highly reasonable.

The most commonly used MRP technique is the dynamic susceptibility contrast (DSC) imaging that requires gadolinium administration, which may lead to some serious fetal adverse effects. As other methods of reperfusion treatment patient selection are easily accessible, DSC-MRP should probably not be used during pregnancy. Clinical DWI, DWI/FLAIR or CTP mismatch could serve this purpose in cases of EVT, IVT or both treatment modalities, respectively. It is also reasonable to utilize arterial spin labeling (ASL) MRI as a noninvasive, non-contrast technique that enables not only cerebral blood flow (CBF), but also occlusion site and collateral flow evaluation [45]. In a systematic review by Liu et al. the agreement on several metrics (hypoperfusion, hyperperfusion, mismatch, and reperfusion) for ASL and DSC was concluded to be moderate to very high, with some heterogeneity of perfusion parameters in analyzed studies [46]. ASL-MRP is a valuable imaging method in certain populations with contraindications for contrast administration, including childbearing women in cases of AIS.

It seems reasonable to forfeit perfusion imaging in AIS pregnant patients presenting within the reperfusion treatment window and proceed to appropriate management immediately after non-contrast and cerebral vessel imaging. Patients presenting after 6 h of symptom duration and otherwise eligible for EVT should probably have CTP performed when non-contrast CT and CTA were initially selected. If MRI and MRA were chosen, the clinical DWI mismatch or ASL-MRP should be used to determine EVT eligibility, rather than proceeding to DSC-MRP. In view of new data from the CLEAR trial, non-contrast CT evaluation may be sufficient for EVT qualification in the extended window in LVO patients. For this purpose the Alberta Stroke Program Early CT Score (ASPECTS) threshold of ≥ 6 might be used, as applied in most centers participating in this study [39].

Pregnant AIS patients presenting after 4.5 h and before 9 h of last seen well and otherwise eligible for IVT should probably have CTP performed to determine tissue status and potentially initiate treatment. When symptoms are noticed on awakening, DWI-FLAIR mismatch on non-contrast MRI or CTP may be used to determine IVT eligibility. Although not being confirmed by solid evidence, this approach appears to be reasonable, given the very low probability of serious fetal adverse effects and potentially devastating consequences of untreated disabling stroke.

## Intravenous Thrombolysis

The use of IVT for AIS has not been evaluated in pregnant women, as they were excluded from all prospective clinical trials that assessed its efficacy and safety; however, alteplase is a large 59,000 Dalton molecule that does not cross the human placenta and there is no evidence for it to have any teratogenic or mutagenic effects on animal models [47, 48]. It also has a very short plasma half-life of 4–5 min, with only 10% of the administered dose remaining in circulation after 20 min [49]. In the 2018 systematic review by Sousa Gomes et al. results of treatment in 141 pregnant women with various thrombotic events were reported. Among them, 4 maternal deaths (2.8%), 12 major bleeding episodes (8.5%), 13 mild/moderate bleeding episodes (9.2%), 2 fetal deaths (1.4%), 1 child death (0.7%), 9 miscarriages (6.4%) and 14 preterm delivery (9.9%) were noted. There were, however, no maternal or fetal deaths stated to be due to thrombolytic treatment (as a complication of a major bleeding), poor results tended to be reported in older papers, where a substantial number of patients had streptokinase administered [50]. Moreover, complication rates of thrombolysis in pregnant women with various thromboembolic conditions, including AIS, were found to be similar to non-pregnant women by some authors [51]. In 20 published cases of childbearing women receiving IVT for AIS (alone or in combination with EVT) in 2 sICH (with favorable outcome) were noted, and one developed intrauterine hematoma. Other pregnancy complications in those cases were thought to be unrelated to IVT [52]. Therefore, it appears that added risk of thrombolysis during pregnancy could be considered as rather low.

Many authors proved that IVT during pregnancy should not be withheld solely due to pregnancy [49, 52, 53]. In recently published “European Stroke Organisation guidelines on stroke in women: Management of menopause, pregnancy and postpartum” several PICO (population, intervention, comparator, outcome) questions were developed and addressed, including “In pregnant women with acute ischaemic stroke, does intravenous thrombolysis (IVT) improve outcome as compared to no IVT?”. Although no evidence-based recommendation could be made (due to lack of solid data), the majority of expert panel members suggested that “pregnant women with acute disabling ischaemic stroke, who otherwise meet eligibility criteria, can be treated with IVT after appropriately assessing the benefit/risk profile on an individual basis” [54].

In a survey on treatment of acute stroke in pregnant and postpartum women 12 (34%) out of 35 respondents (Canadian stroke practitioners) had an experience with reperfusion treatment and none reported any treatment-related complications. Of those who had never encountered such a scenario 2 (8%) stated that they would always proceed with IV thrombolysis in the case of an otherwise IV-tPA eligible pregnant patient and the remaining 22 (88%) would do it “in some instances” [55]. This reflects the AHA/ASA guideline statement that IVT can be considered in pregnancy when benefits of treating moderate or severe stroke outweigh the risk of uterine bleeding [7]. According to Canadian Stroke Best Practice Consensus Statement “treatment options […] should promptly be considered in consultation with an interdisciplinary team with expertise in neurology, obstetrics and gynecology, maternal-fetal medicine, and interventional radiology, where possible and available.” [12]. It appears to be a very reasonable approach, although it may lead to a substantial treatment delay. Considering the rather low added risk of serious IVT complications, we feel that decision should be fairly swift and tilt in favor of treatment unless well-known contraindications are present. When eligible, intravenous alteplase dose of 0.9 mg/kg (maximum of 90 mg) adjusted to the prepregnancy or early pregnancy weight should probably be administered [53].

Understandably, no childbearing woman was included in trials assessing IVT in late presenting and undetermined onset AIS patients; however, there is no reason to assume that changing the treatment eligibility paradigm from time to tissue based would not apply to this subgroup. Therefore, in view of previously mentioned data on various imaging modalities (in the “Perfusion imaging and late presenting/unknown onset AIS patient” section) and alteplase safety, it may be reasonable to proceed with an appropriate protocol of extended window IVT selection in pregnant patients provided the AHA/ASA requirement of predicted benefits outweighing the risk of uterine bleeding is met.

## Mechanical Thrombectomy

The use of MT has proved to be a very efficient treatment method in carefully selected AIS patients [7]. Similar to IVT, it has not been evaluated in childbearing women due to relevant RCTs exclusion criteria. The primary concerns of EVT during pregnancy may be treatment-related radiation, contrast media exposure and potential complications of anesthesia. The risk of additional contrast media administration is probably minor as described earlier.

In an experiment using standard body phantom by Marshman et al. the uterine radiation dose was estimated to be 0.011 mSv/min and 4.6 mSv/min for scattered and direct exposure, respectively. Given the approximate time of 30 s of groin and 45 min of head irradiation, the predicted fetal dose was 2.8 mSV [56]. In a more recent analysis of 3 pregnant patients undergoing MT for AIS the whole-body effective dose of CT and EVT was measured to be 13.2 ± 0.67 mSv (range 11–22.3 mSv). The estimated dose received by fetuses in this study was 0.024 ± 0.018μSv (range 0.0026–0.060 μSv). The authors concluded that fetal radiation exposure during EVT is equivalent or even lower than other emergency diagnostic imaging studies, such as CT pulmonary angiogram and whole-body CT in trauma patients [57]. It is important to note that these doses are far below estimated threshold for fetal adverse events [9, 11, 14–17]. Nevertheless, several interventions to further reduce the radiation exposure including lead apron for shielding of uterus, minimizing number of DSA exposures, and low-dose pulsed fluoroscopy utilization should probably be introduced [57]. Direct thromboaspiration technique may also shorten both procedural and fluoroscopy time comparing to stent-retriever thrombectomy under continuous distal aspiration that may be beneficial in pregnant patients [58].

The functional outcomes of MT under general anesthesia (GA) are similar to those performed under conscious sedation (CS) in non-pregnant patients [59]; however, GA poses a risk of hypotension and strict blood pressure control protocol should be utilized to avoid its adverse events that may potentially compromise the uteroplacental perfusion [60, 61]. Cautious use of analgesics and sedatives, as well as proper positioning to avoid aorto-caval compression regarding the duration of gestation are of great importance [62]. CS or local anesthesia (LA) might be preferable when feasible.

In a recent analysis of US national claims data, 4590 pregnant and postpartum AIS patients were identified, with 180 (3.9%) treated by means of MT. Compared to nonpregnant patients, MT-treated women showed lower rates of intracranial hemorrhage (11% versus 24%, *P* = 0.069) and poor functional outcome defined as maternal death or intermediate and long-term care/nursing facility discharge (50% versus 72%, *P* = 0.003). There was no mortality or miscarriage during hospitalization after EVT noted [63]. Data from this study and several case reports suggest that MT is safe and efficacious treatment of AIS due to LVO during pregnancy [8, 64, 65]. This view is reflected by the previously cited Canadian survey on treatment of acute stroke in pregnant and postpartum women. Among 25 respondents who had never encountered a scenario of childbearing woman eligible for MT, 19 (76%) would proceed with treatment without other requirements and remaining 6 would introduce EVT “in some instances” focusing primarily on stroke severity and risk-benefit ratio, with a lesser emphasis on the consent process [55]. In 2022 ESO guidelines on stroke in women all of the expert panel members suggested that “pregnant women with acute ischemic stroke and large vessel occlusion, who otherwise meet eligibility criteria, can be treated with MT after appropriate assessment of the benefit/risk profile on an individual basis” [54].

Utilization of bridging therapy with IVT in AIS patients eligible for both methods of reperfusion therapy is now a subject of wide consideration. In a recent meta-analysis of RCTs assessing direct MT versus bridging therapy showed no difference in improving good functional outcome [66]. Considering these data, in EVT-eligible childbearing women it is reasonable to avoid additional treatment risk by deferring IVT and proceeding directly to MT when readily available. This view was confirmed by a majority of expert committee members in ESO guidelines and shared by most of stroke practitioners in one study [54, 55].

Considering unequivocal evidence on MT benefits in appropriately selected late presenting and undetermined onset AIS patients, there is no reason to exclude pregnant women from this highly beneficial treatment (as argued in “Perfusion imaging and late presenting/unknown onset AIS patient” and “Intravenous thrombolysis” sections) [37, 38].

In conclusion, pregnancy should not be a contraindication for MT in otherwise eligible AIS patients as stated in some guidelines [12, 54]. This should probably include childbearing women presenting beyond 6 h from last seen well. Withdrawing pregnant patient from IVT in cases of being eligible for EVT when it is readily available might be considered in some instances (i.e. LVO AIS patient presenting to comprehensive stroke center).

## Conclusion

Managing AIS stroke during pregnancy may be very challenging. Given the serious prognosis and a potential to significantly improve both maternal and fetal outcome, prompt and adequate decisions concerning neuroimaging and reperfusion treatment should be made. In such instances, the initial non-contrast imaging method (CT or MRI) is preferably chosen based on its availability, rather than fetal safety concerns. Vascular imaging should then be rapidly introduced according to previous imaging modality selected in individuals otherwise eligible for endovascular treatment (EVT). Some preselection methods based on National Institutes of Health Stroke Scale (NIHSS) may be introduced. Due to rather low added risk of serious treatment complications, pregnancy should not be a sole contraindication for neither intravenous thrombolysis (IVT), nor EVT. It is also reasonable not to exclude otherwise eligible late presenting and unknown onset childbearing AIS women from reperfusion treatment. Additional safety measures, such as appropriate shielding of the abdomen/pelvis and minimizing imaging scans when performing diagnostic or treatment procedures utilizing x‑ray should be routinely taken.
